# Pyroelectric Energy Conversion and Its Applications—Flexible Energy Harvesters and Sensors

**DOI:** 10.3390/s19092170

**Published:** 2019-05-10

**Authors:** Atul Thakre, Ajeet Kumar, Hyun-Cheol Song, Dae-Yong Jeong, Jungho Ryu

**Affiliations:** 1 School of Materials Science and Engineering, Yeungnam University, Gyeongsan 38541, Korea; atulthakre09@gmail.com (A.T.); jkajeet@yahoo.co.in (A.K.); 2 Center for Electronic Materials, Korea Institute of Science and Technology (KIST), Seoul 02792, Korea; hcsong@kist.re.kr; 3 Department of Materials Science & Engineering, Inha University, Incheon 22212, Korea; 4 Institute of Materials Technology, Yeungnam University, Gyeongsan 38541, Korea

**Keywords:** pyroelectric materials, thermal energy harvesters, flexible

## Abstract

Among the various forms of natural energies, heat is the most prevalent and least harvested energy. Scavenging and detecting stray thermal energy for conversion into electrical energy can provide a cost-effective and reliable energy source for modern electrical appliances and sensor applications. Along with this, flexible devices have attracted considerable attention in scientific and industrial communities as wearable and implantable harvesters in addition to traditional thermal sensor applications. This review mainly discusses thermal energy conversion through pyroelectric phenomena in various lead-free as well as lead-based ceramics and polymers for flexible pyroelectric energy harvesting and sensor applications. The corresponding thermodynamic heat cycles and figures of merit of the pyroelectric materials for energy harvesting and heat sensing applications are also briefly discussed. Moreover, this study provides guidance on designing pyroelectric materials for flexible pyroelectric and hybrid energy harvesting.

## 1. Introduction

Energy has been a prime concern of the scientific community and industrial areas worldwide. As the demand for self-powered autonomous electronics with low power consumption has grown drastically in the electronics industry, energy harvesters have received immense focus and are being widely studied. Solid-state batteries, which are commonly used as an energy source in devices, require frequent and periodic maintenance such as recharging or replacement. Thus, various scavenging methods (harvesting the energy from stray energy sources) have been proposed to provide sustainable energy supplies for small electronic devices [1,2,3,4,5,6,7,8,9,10]. For example, piezoelectric energy harvesters from mechanical energy [2], thermal energy harvesters utilizing temperature differences through thermoelectricity or pyroelectricity [11], magnetic energy harvesters utilizing the stray magnetic noise from magneto-electric properties [12], and solar cells using the photovoltaic effect from solar light are areas of focus [13].

Among the above forms of stray energy, heat is ubiquitous and serves as a low-grade waste [14]. To convert thermal energy into usable electricity, thermoelectricity and pyroelectricity can be utilized. Thermoelectric materials have been employed to convert the spatial thermal gradient into electrical energy, i.e., the Seebeck effect [11]. Meanwhile, pyroelectricity is a phenomenon in which temperature fluctuations in the environment are converted into electrical energy [15]. Pyroelectric materials need a temporal temperature gradient just as thermoelectric materials need a spatial gradient [11,16]. Variation in the temperature of the pyroelectric material causes a net dipole moment, which further results in the accumulation of charges at the electrode, separating application targets such as small scale microgenerators with small dimensions (small enough for spatial temperature fluctuations). Thermoelectric materials have a lower ZT (“Z” is Ioffe’s figure of merit) at room temperature. Thus, pyroelectric energy harvesting (PyEH) is preferable for harvesting low-grade thermal energy and at low temperatures.

**Figure 1:**
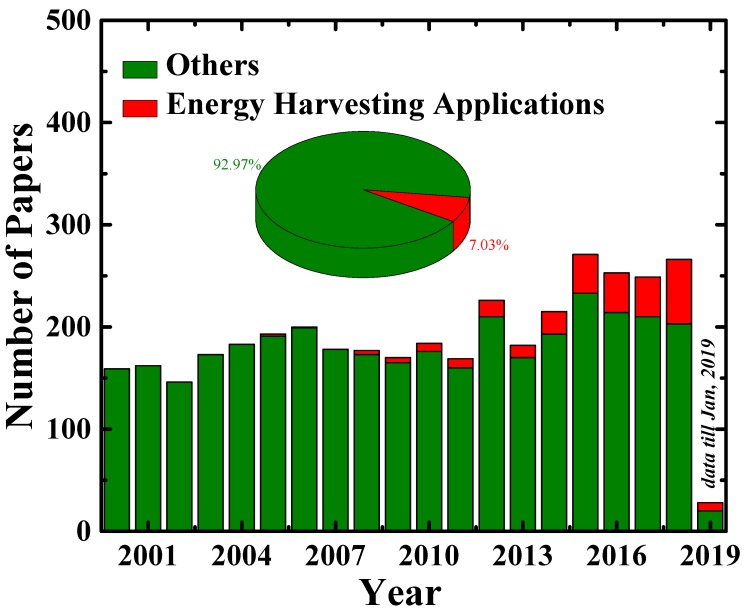
The histogram distribution of the number of papers on the pyroelectric materials published in the last two decades. The inset image shows a pie chart for the number of papers reporting the application of thermal energy harvesters (red) through pyroelectricity. The data have been taken from “Web of Science” database (https://www.webofknowledge.com), and the actual data may vary from the data shown here.

The typical application arena of pyroelectric energy conversion is a thermal sensor, which can detect thermal signals at the moment. However, it can also be used as a thermal energy harvester if the conversion efficiency and total converted energy are sufficiently high to charge electrical energy storage devices, such as a supercapacitor or battery. Although the PyEH concept was introduced in the 1960s, it still remains a comparatively less studied area [17,18,19,20,21,22,23]. The reports estimate that in 2009, over 50% of the total consumed energy was wasted as heat, which is mostly from electrical power generation and automobile systems [24]. In addition, a large amount of heat energy is lost through electrical appliances, such as refrigerators, air conditioning systems, and heat pumps. Although the study of PyEH accounts for a just small fraction of the total amount of studies on pyroelectric materials, it can be noted from Figure 1 that over the past few years, the number of research articles focused on it has increased. Further study of PyEHs would clearly benefit the utilization of this wasted heat.

In the 21st century, wearable and implantable electronics have gained considerable attention [25,26,27,28,29,30,31,32,33]. To power these electronics, which are necessarily small, flexible, and endurable, onboard power sources are required. As with other autonomous devices, PyEHs could be the optimal powering solution for such devices. Several reports have demonstrated significant generated output power densities (in the range of ~ µW∙cm^−3^ to mW∙cm^−3^) using pyroelectric energy conversion, which can be used to drive devices, such as liquid color displays (LED), light-emitting diodes (LED), and wireless devices [34,35]. Among various energy harvester candidates, although PyEHs have great potential for such applications, they are the least explored area. In addition, energy harvesters are clearly necessary for the flexibility of wearable and implantable devices. Many scientific groups have reported various demonstrations of flexible pyroelectric or hybrid pyroelectric–piezoelectric energy harvesters in recent years [36,37,38,39,40,41,42,43,44,45,46].

Over the past two decades, many pyroelectric materials have been extensively studied, as shown in Figure 1, with significant experimental findings for energy harvesting application having been reported. Few reviews have also been published in which several pyroelectric materials and the methods to harvest the thermal energy have been addressed. A historical review of pyroelectricity has been reported by Lang et al. [47]. Pyroelectric materials and the methods of energy harvesting have also been covered in previous reviews [14,48,49]. This review intends to address the concept, figures of merit of pyroelectricity, and recent progress in pyroelectric materials, such as ferroelectrics and relaxor ferroelectrics, efficient methods to harvest thermal heat fluctuations, and flexible and hybrid energy harvesters based on pyroelectricity. Moreover, this study provides guidance toward designing pyroelectric materials for applications of flexible pyroelectric energy harvesters (PyEH) and hybrid energy harvesters (piezo–pyro energy harvester).

## 2. Theory of Pyroelectricity

### 2.1. Concept of Pyroelectricity

Depending on electrical conduction properties, typically two different kinds of properties can be employed to convert the thermal gradient into electrical energy, pyroelectricity (e.g., insulator) and thermoelectricity (e.g., Seebeck effect for a semiconductor or metal). In general, thermoelectricity results from four different processes—the Seeback effect, Peltier effect, Joule heating, and Thomson effect—in which electrons from a hot zone with higher energy flow into a cold zone to mitigate the thermal energy difference. As an electron has an electrical charge, electrical current is simultaneously generated because of the thermal energy gradient. Here, as an electron flows inside the material along a certain direction, a semiconductor, which allows electron flows with a low resistance at a certain temperature difference on account of the energy bandgap, should be applied. In the case of a thermal gradient, i.e., a stable temperature difference, continuous electron flow is also maintained, resulting in a direct current (DC). Meanwhile, pyroelectricity is a phenomenon in which the temperature fluctuations of pyroelectric material induce a change in polarization change, which further causes the separation of bound charges [50]. Here, special attention should be paid to the term “temperature fluctuation”: it refers to the dynamic condition in which temperature changes with time. As such, pyroelectricity can result in an alternating current (AC).

Pyroelectric materials are the subdomain of dielectrics which have polar symmetry, and they exhibit a spontaneous polarization (*P_s_*) when there is no applied electric field. In general, polarization is caused by the crystalline structure in the ionically bonded material. In the case of the polymers, polarization can arise because of the alignment of the covalent bonded atoms in the molecular chain [51]. The fluctuation in the temperature causes a change in the polarization and corresponding electric current.

Assuming there is a pyroelectric material with a temperature gradient *dT*, then the polarization change *dP* occurring in materials and pyroelectric coefficient *λ* can be defined as

(1) $$\lambda^{\sigma ,E}={\left(\frac{dP_{s}}{dT}\right)}_{\sigma ,E}=\frac{dQ}{SdT}$$

where *σ* and *E* are the constant stress and constant electric field, respectively. *Q*, *T*, and *S* are the induced charge, temperature, and surface area of the electrode, respectively. *P_s_* is the electrical polarization of the pyroelectric material sandwiched between two metal electrodes, forming a parallel-plate capacitive structure with the poling direction normal to the electrode plates and cross-sectional area *S*.

When the pyroelectric material is heated, i.e., *dT/dt* > 0, then the thermal vibration causes a disturbance in the dipole alignment and, therefore, *P_s_* changes. The significant amount of change in the *P_s_* results in the separation of bound charge carriers and the accumulation of the charges at the electrode surface. Also, when the pyroelectric material is cooled, i.e., *dT/dt* < 0, The dipoles realigns themselves which further results in an enhancement in the *P_s_*. When the pyroelectric capacitive structure is connected to the load, a corresponding electric current flows through the load [49]:

(2) $$I=\frac{dQ}{dt}=S\lambda \frac{dT}{dt}$$

where *t* is time. The total accumulated charge can be represented as the integral of Equation (2) as shown below.

(3) $$Q=\int_{t_{i}}^{t_{f}}Idt=\int_{t_{i}}^{t_{f}}S\lambda \frac{dT}{dt}dt=\int_{T_{i}}^{T_{f}}S\lambda dT=S\lambda \left(T_{f}-T_{i}\right)$$

where subscripts *i* and *f* represent the initial and final conditions, respectively. Here, it can be noted that the accumulated charge does not depend on the rate of change of the temperature but on the difference between the initial and final temperature. Meanwhile the pyroelectric current directly depends on the rate (time) of change of the temperature. Thus, the larger the pyroelectric coefficient is, the larger the electrode area is, and large temperature fluctuations with time would result in large separation of bound charges. In addition, it can be inferred from Equation (1) that the pyroelectric coefficient does not depend on the distance between the electrode layers [52].

Because Equation (1) implies that the pyroelectric coefficient strongly depends on the *P_s_* of the material, a significant level of polarization needs to be present. As we categorize the different types of dielectrics, all pyroelectrics are also piezoelectric and, similarly, all ferroelectrics are also pyroelectric. Therefore, all ferroelectrics are pyroelectric and piezoelectric. Previous reports have indicated that ferroelectric materials tend to exhibit a higher piezoelectric and pyroelectric coefficient than non-ferroelectric materials. However, pyroelectricity and piezoelectric properties vanish when ferroelectric materials are heated above the transition temperature (*T_c_*). *P_s_* also decreases rapidly beyond *T_c_*. Similarly, *dP_s_/dT* initially increases until the temperature reaches *T_c_*, and then drops to zero at *T_c_*, i.e., pyroelectricity is no longer present beyond *T_c_*. It can be noted that the pyroelectric coefficient has a maximum value, and the corresponding accumulated charges would be highest just before *T_c_*. In Table 1, some of the reports of pyroelectric thermal energy harvesting in ferroelectric materials (reported mainly over the last five years) are listed, which we will be discussing in this review in the subsequent sections.

**Figure 2:**
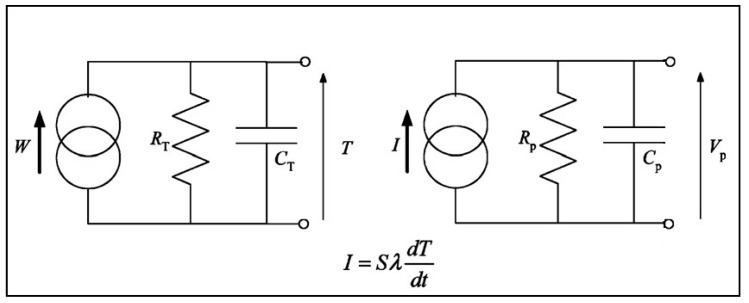
Thermal and electrical equivalent circuit of a pyroelectric cell (reproduced with permission) [53].

The pyroelectric capacitive structures are generally modeled as current sources. Figure 2 shows the general electric lumped element circuit model of a pyroelectric system [53]. In this theoretical equivalent circuit of general pyroelectric transducer *W* represents the incident thermal power. The *I* and *V_p_* are induced current (pyroelectric current) and peak–peak open circuit output voltage. *R_T_*, *C_T_*, *R_P_*, and *C_P_* represent the thermal resistance and capacitance, and the electrical resistance and capacitance, respectively [54].

### 2.2. Figure of Merit

The pyroelectric effect has been extensively studied for various applications, such as infra-red sensors, thermal imaging or intruder alarms, gas sensors, and fire alarms. Thus, for the proper selection of materials for these applications, figures of merit (FoM) for thermal energy conversion have been suggested in the literature [55,56,57]. Specifically, for application as sensors, high voltage and high current are desired for a given stimulation [52]. In addition, for the thermal energy harvesting applications, an electrothermal coupling factor in addition to generated current and voltage responsivities have been suggested to predict the efficiency of the material. In this section, the FoMs of pyroelectric materials for heat sensing and energy harvesting applications will be discussed.

The current responsivity of the pyroelectric materials is defined as

(4) $$F_{i}=\frac{\lambda }{c_{E}}=\frac{\lambda }{\rho c_{P}}$$

where *c_E_* and *c_P_* are the volume specific heat (J∙m^−3^∙K^−1^) and specific heat capacity, respectively. The density of the material is given by *ρ* (kg∙m^−3^). Similarly, the voltage responsivity can be defined as

(5) $$F_{v}=\frac{\lambda }{c_{E}\varepsilon_{33}^{\sigma }}=\frac{\lambda }{\rho c_{P}\varepsilon_{33}^{\sigma }}$$

where $\varepsilon_{33}^{\sigma }$ is the dielectric permittivity in the polarization direction at constant stress *σ*. The current and voltage responsivities (*F_i_* and *F_v_*, respectively) are often used for the selection of the materials for different sensor applications. However, in the case of energy harvesting applications, where the converted energy, output power, and the efficiency (for the conversion from thermal to electrical energy) are important additional key criteria, the electrothermal coupling factor is the most critical key FoM. It can be defined as

(6) $$k^{2}=\frac{\lambda^{2}T_{hot}}{c_{E}\varepsilon_{33}^{\sigma }}=\frac{\lambda^{2}T_{hot}}{\rho c_{P}\varepsilon_{33}^{\sigma }}$$

where *T_hot_* is the maximum operating temperature [58]. The efficiency of the pyroelectric thermal energy harvesting can be directly estimated by the FoM. Most pyroelectric materials have a thermal coupling coefficient value < 1%. Apart from *k*^2^, another PyEH FoM, i.e., *F_E_*, has also been suggested, and can be defined as [59]

(7) $$F_{E}=\frac{\lambda^{2}}{\varepsilon_{33}^{\sigma }}$$

As it can be noted, the energy harvesting FoM does not depend on the heat capacity of the pyroelectric material, whereas the current and voltage do. In addition, the presented FoMs are the static definitions and do not consider the transient nature of the heat transfer and dielectric losses [50]. The FoM, which considers dielectric loss and heat diffusion, has been suggested and can be employed for the energy harvesting applications [51].

## 3. Pyroelectricity in Ferroelectric Materials

### 3.1. Cycles of Pyroelectric Energy Harvesting

**Figure 3:**
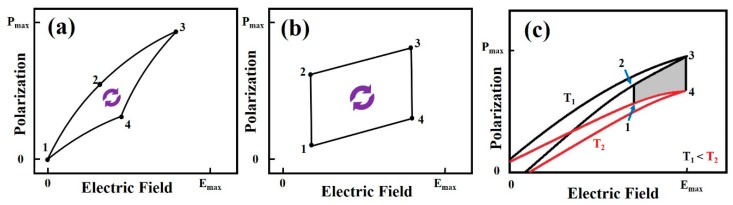
Thermodynamic cycles showing pyroelectric energy harvesting (PyEH) efficiency. (**a**) Carnot, (**b**) Ericson, and (**c**) Olsen cycle. (P_max_ and E_max_ are the maximum polarization and maximum applied electric field, respectively.)

To elucidate the PyEH efficiency, there are a few thermodynamic cycles, such as the Carnot cycle, Ericson cycle, and Olsen cycle, as shown in Figure 3, that have been proposed [17,19,22]. In subsequent sections, these thermodynamic cycles of energy conversion are briefly discussed. In this review, we focus on the PyEH through the Olsen cycle.

#### 3.1.1. Carnot Cycle

The Carnot cycle of energy conversion is an efficient thermodynamic cycle, which consists of two isothermal (path 1 to 2) and two adiabatic (path 3 to 4) processes, as shown in Figure 3a. Here, state “1” denotes the initial state of the pyroelectric material at Curie temperature *T_c_* when no electric field is applied. Now, an external electric field is applied, and the material goes into state “2” through the isothermal process, i.e., the temperature remains the same. Similarly, the applied electric field is further increased, and an adiabatic process takes place from state “2” to “3,” i.e., no heat is exchanged from the environment. The applied electric field in state “3” is kept lower than the dielectric breakdown strength of the pyroelectric material at the operating temperature. Now, the applied electric field is decreased for an isothermal process, which makes a state transition from “3” to “4.” In continuation, the applied electric field is further reduced to zero, and an adiabatic process takes place, in which the material returns to the initial state, i.e., “1.” Here, the efficiency of the energy conversion ($\eta_{Carnot}$) between two reservoirs can be expressed as

(8) $$\eta_{Carnot}=1-\frac{T_{c}}{T_{h}}$$

where *T_c_* and *T_h_* are the temperatures of the hot and cold reservoirs, respectively.

Although, the Carnot cycle is considered the most efficient cycle, it also has some limitations, such as the requirement of two adiabatic and two isothermal processes. These limitations make the Carnot cycle difficult to employ; hence, it is often used only for comparison with other cycles.

#### 3.1.2. Ericson Cycle

The pyroelectric Ericson cycle for the energy conversion was first introduced by Olsen in 1980 [60]. The Ericson energy conversion cycle comprises two constant electric field processes (transition from state “1” to “2” and “3” to “4”) and two isothermal processes (transition from state “2” to “3” and “4” to “1”), as shown in Figure 3b. Just as in the Carnot cycle, state “1” represents the initial state of the pyroelectric material at temperature *T_h_* when no external applied electric field is applied. Then, the material is cooled until *T_c_* of the material is reached, i.e., state “2.” Furthermore, an isothermal process takes place with an increasing external electric field until *E_max_* is achieved, as the material approaches state “3.” Now, at this constant electric field, the material is heated until *T_h_* is reached, as the material enters state “4.” Finally, this process is followed by an isothermal process, which takes place when the applied electric field decreases to zero while the temperature remains constant, i.e., *T_h_*. The electrical work, *W_Ericson_*, in this cycle can be expressed as

(9) $$W_{Ericson}=-(T_{h}-T_{c})\int_{0}^{E_{\max}}pdE$$

The heat inflow from the reservoir can be expressed as

(10) $$Q_{in}=c(T_{h}-T_{c})+\int_{0}^{E_{\max}}pT_{h}dE$$

Therefore, the efficiency of the Ericson cycle ($\eta_{Ericson}$) can be expressed as

(11) $$\eta_{Ericson}=\frac{\left|W_{cycle}\right|}{Q_{in}}=\frac{\int_{0}^{E_{\max}}pdE}{c+\frac{T_{h}}{\left(T_{h}-T_{c}\right)}\int_{0}^{E_{\max}}pdE}$$

#### 3.1.3. Olsen Cycle

The thermodynamic Olsen cycle is a modified Ericson cycle. The Ericson cycle does not include the hysteresis loss. Olsen suggested this cycle between two polarizations vs. electric field (*P*–*E*) hysteresis curves for a pyroelectric material at two different temperatures (T_1_ and T_2_) for the pyroelectric energy conversion. As shown in Figure 3c, the dark shaded area, enclosed by the path 1-2-3-4, represents the electrical work, *W_Olsen_*, in this cycle. In Table 1, a comparison of energy densities using the Olsen cycle for various reported pyroelectric materials has been provided. In general, the Olsen cycle is quite popular owing to its efficiency and ease of employability in pyroelectric materials.

### 3.2. Pyroelectric Materials

Several pyroelectric materials have been widely explored for thermal energy harvesting applications [59,61,62,63,64,65,66,67,68,69,70,71,72,73,74,75,76]. As the inset of Figure 1 shows, the study of thermal energy harvesting application of pyroelectric materials makes up a very small percentage of the total number of studies on pyroelectric materials reported in the last two decades. In the quest for the energy harvesting applications, ferroelectric materials in different forms such as triglycine sulfate (TGS), polymers (polyvinylidene fluoride; PVDF), bulk perovskite ceramics, single-crystals, and its thick and thin films have been extensively exploited [51]. TGS possesses a very high pyroelectric coefficient, but it is fragile, water soluble, and tends to decompose in high humid or vacuum conditions. Therefore, it becomes difficult to employ in energy harvesting device applications, but it is useful in sensing applications such as thermal imaging [77,78].

For the sensors and device applications, ferroelectric polymers and its composites have drawn the attention of the scientific community owing to its various functionalities such as flexibility and easy fabrication [79]. Although polymers such as PVDF possess poor pyroelectric coefficient, they are very suitable for application owing to their easy fabrication for large area thin films; greater stability than TGS when subjected to heat, vacuum, and moisture; and mechanical robustness [51,80]. Although, PVDF has low heat conductivity and low permittivity, it has been widely reported and employed as sensors in devices such as burglar alarms [81]. The perovskite-structured ferroelectric ceramics are more popular than the polymer for the PyEH applications. These ceramics are robust in nature and remain stable in high humid or vacuum conditions and are thermally more stable than polymers. In addition, they possess a high pyroelectric coefficient and low dielectric loss, which makes them suitable for energy harvesting applications. Because the pyroelectric properties of these ceramic materials depend on its composition, many of these ceramics have been engineered to enhance the maximum harvestable energy density [50]. In a broad sense, for small-area application, the materials exhibiting a large dielectric constant such as perovskite-structured ferroelectric ceramics are preferred, whereas for large-area application, materials exhibiting a low dielectric constant such as PVDF are preferred.

#### 3.2.1. Lead-Free Pyroelectric Materials

**Figure 4:**
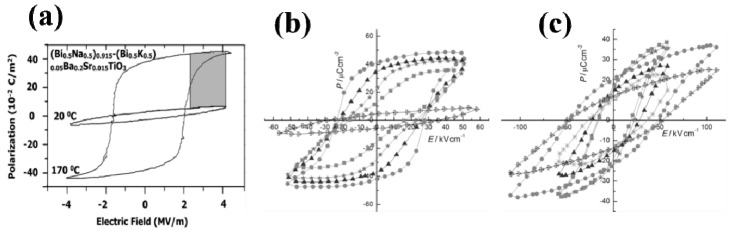
(**a**-**c**) shows the *P*–*E* hysteresis loops for BNT-BKT-BT-ST, BNKT, and BNLT ceramics, respectively, at different temperatures (reproduced with permission) [84,86].

Among the ferroelectric ceramics, lead-free perovskites are potential candidates for energy harvesting applications owing to their excellent pyroelectric properties and environment compatibility. In addition, it can be noted from Table 1 that the lead-free pyroelectric exhibits a higher energy density at a low applied electric field than the lead-based ceramic pyroelectric. Bismuth sodium titanate-barium titanate (BNT-BT) ceramics have been quite popular and are considered a potential candidate for PyEH materials owing to its excellent piezoelectric and pyroelectric coefficients, and high *T_c_* (593 K) [95]. On the contrary, BNT-based ceramics are difficult to use because of their high electrical conductivity and dielectric loss. Therefore, many studies on pyroelectric characteristics have been published based on engineered or doped-BNT ceramics [96,97,98,99,100,101,102,103,104]. Vats et al. reported great thermal energy harvesting in a lead-free (Bi_0.5_Na_0.5_)_0.915_(Bi_0.5_K_0.5_)_0.05_Ba_0.02_Sr_0.015_TiO_3_ ceramic using the Olsen cycle [84], as shown in Figure 4a. In this report, the thermal energy density was studied with different temperature ranges, and the maximum energy density for the ceramic is estimated at 1523 k∙J∙m^−3^∙cycle^−1^ in the temperature range of 193 to 433 K with electric field of 1–40 kV∙cm^−1^. The authors observed the decrease in the energy density in the temperature range above 433 K and attributed it to the ferroelectric- to antiferroelectric-like transition of the ceramic. The reported energy density value was higher than the values reported for various pyroelectric materials such as lead-based ceramics (e.g., (Pb_x,_La_1-x_)ZrTiO_3_ and Pb(Mg,Nb)O_3_-PbTiO_3_) and polymers (polyvinylidene difluoride-trifluoroethylene (PVDF-TrFE)) [63,64,65,70,71,73,105,106,107,108]. In another report from the same group, alkali-substituted, i.e., K- and Li-doped, BNT was also studied with two different compositions, i.e., Bi_0.5_Na_0.44_K_0.06_TiO_3_ (BNKT) and Bi_0.5_Na_0.425_Li_0.075_TiO_3_ (BNLT), for thermal energy harvesting purposes [86]. The authors obtained the energy densities of 1986 kJ∙m^−3^∙cycle^−1^ (298–393 K and 1–52 kV∙cm^−1^) in BNKT and 1146 kJ∙m^−3^∙cycle^−1^ (298–383 K and 1–112 kV∙cm^−1^) in BNLT. Here, it can be noted that BNKT requires a smaller electrical field (52 kV∙cm^−1^) than BNLT (where *E_H_* is 122 kV∙cm^−1^) to achieve saturation polarization, which makes the BNKT superior for thermal energy harvesting applications. The authors also observed here that the change in the *E_L_* does not affect the energy harvesting in BNLT as much as in BNKT, due to the steeper *P*–*E* loop of BNKT, as shown in Figure 4b,c. Therefore, BNLT has superior practical potential application as a thermal energy harvester as the transfer requirement would be low (due to the low difference in the applied electrical field).

**Figure 5:**
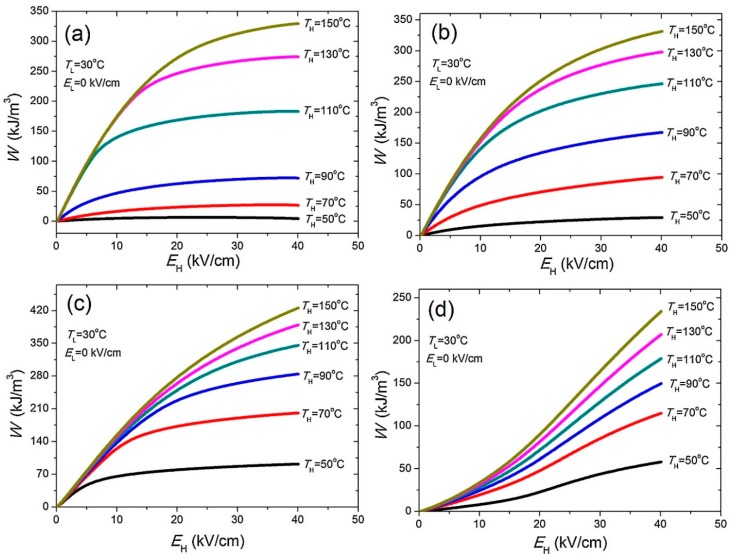
The measured pyroelectric energy harvesting performance of NBBSTx ceramic with different compositions (**a**) 0.10, (**b**) 0.15, (**c**) 0.20, and (**d**) 0.25 (reproduced with permission) [68].

In both reports mentioned above, the BNT-BT-based lead-free ceramics exhibit a remarkable energy density, higher than that of lead-based ceramics reported up to now. This makes these lead-free ceramics strong candidates for application as thermal energy harvesters. In addition, some other lead-free ceramics composites were also studied for thermal energy harvesting by the same Vaish et al. group, such as KNTM, i.e., K[(Nb_0.90_Ta_0.10_)_0.99_Mn_0.01_]O_3_, and BZT-50BCT, i.e., 0.5Ba(Zr_0.2_Ti_0.8_)O_3_-0.5(Ba_0.7_Ca_0.3_)TiO_3,_ in which the maximum harvestable energy density 629 kJ∙m^−3^∙cycle^−1^ was obtained using the Olsen cycle [83]. Apart from these ceramics, Na_0.5_Bi_0.5_TiO_3_ (NBT)-based ceramics were also explored for the electrocaloric effect (ECE) and PyEH applications [68]. NBT ceramics exhibit large polarization, which makes them a promising candidate for both ECE and PyEH applications. Luo et al. reported a study of the ECE and PyEH properties in NBT-based ceramics with different compositions i.e., (0.94 − *x*)Na_0.5_Bi_0.5_TiO_3_-0.06BaTiO_3_-*x*SrTiO_3_ (NBBST*x*) where *x* = 0.10, 0.15, 0.20, and 0.25 of SrTiO_3_ (ST) insertion. The composition of the insertion of ST into the NBT ceramic strongly affects both the ECE and PyEH characteristics because of the decrease in the ferroelectric-relaxor transition temperature. Here, the PyEH calculations were performed using the Olsen cycle, as shown in Figure 3c. The energy density for the ceramics was calculated as a function of E_h_ and T_h_ while maintaining E_l_ and T_l_ constant at 0 kV∙cm^−1^ and 303 K, respectively, as shown in Figure 5. Here, the NBBST0.20 ceramic exhibited the highest energy density ~425 kJ∙m^−3^∙cycle^−1^ for the EH and TH as 40 kV∙cm^−1^ and 423 K, respectively. This study demonstrates how the tuning of the pyroelectric thermal energy density of the ceramics can be performed with different insertion of ST into the system. In addition, the reported PyEH was higher than the reported PyEH for the conventional lead-based bulk ferroelectrics, such as PNZST (i.e., 300 kJ∙m^−3^∙cycle^−1^) [70], PZN-0.45PT single-crystal (i.e., 242.7 kJ∙m^−3^∙cycle^−1^) [63], and PMN-32PT [64] single-crystal (i.e., 100 kJ∙m^−3^∙cycle^−1^).

The aforementioned studies have demonstrated that the PyEH of lead-free ceramics are higher than that of the typical lead-based ceramics. However, the complex compositions and chemical formulas might hinder mass production from the application perspective. Therefore, ferroelectric materials with simple structures, such as ferroelectric polymers (PVDF-TrFE) and binary oxides (HfO_2_), were explored. Also, the thin films of these ceramics or polymers were extensively explored for PyEH as a result of nanoscale application such as a pyroelectric nanogenerator (PyNG). Yang et al. demonstrated that the PyNGs can harvest thermal energy more efficiently [109]. A PyEH from P(VDF-TrFE) of 521 and 155 kJ∙m^−3^∙cycle^−1^ were reported by Navid et al. [110] and Lee et al. [111], respectively. From the above discussion, it can be summarized that the efficient and environmentally friendly PyEH could have a lead-free composition, thin film or nanostructured form, temperature stability, and high dielectric breakdown strength.

**Figure 6:**
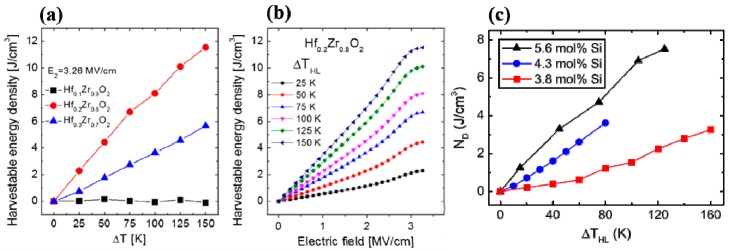
(**a**) The harvestable energy density with regard to ΔT and (**b**) electric field, where T_L_ is kept constant at 298 K for the different compositions of the HZO calculated from *P*–*E* hysteresis loops [87]. (**c**) Harvestable energy density (N_D_) per Olsen cycle with regard to ΔT_HL_ (TL = 298 K) for different mol% of Si (reproduced with permission) [88].

For the miniaturization of the devices, several ferroelectric materials have been proposed as PyEH nanogenerators. Among these, HfO_2_ thin films deposited on Si substrate became quite popular because of its great compatibility with Si for monolithic device applications, such as nanoscale PyEH, electrocaloric cooling, energy storage capacitors, and infrared sensors. The binary oxides have good temperature stability and can be employed for 3D nanostructures using the atomic layer deposition (ALD) technique. Park et al. reported a colossal energy density in the nearly 9.2-nm-thick ALD-deposited Hf_x_Zr_1-x_O_2_ (where x is varied from 0.1 to 0.3) (HZO) on SiO_2_/Si substrate. The Hf_x_Zr_1-x_O_2_ with x = 0.2 and 0.3 exhibited values of 11500 (298–423 K and 0–3260 kV∙cm^−1^) and 5700 kJ∙m^−3^∙cycle^−1^ (298–423 K and 0–3260 kV∙cm^−1^), respectively, which was approximately 7.6 and 3.7 times higher than the largest value reported (Figure 6). The PyEH calculations were conducted using the Olsen cycle. As for the sensor application point of view, the HZO thin films have also shown promising pyroelectric FoM, such as an *F_v_* of 0.32 m^2^∙C^−1^. The great compatibility of HZO with the Si substrate along with the remarkable PyEH and FoMs makes it an excellent choice for device application.

**Figure 7:**
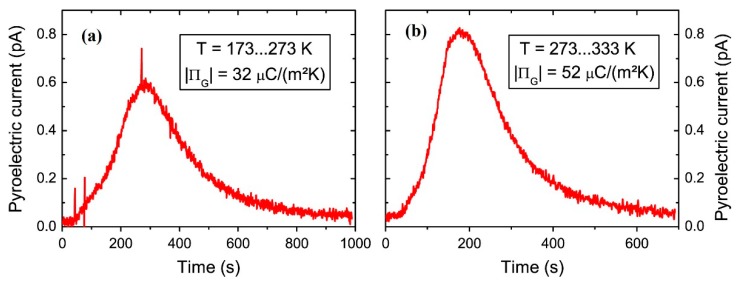
Pyroelectric coefficients (shown in inset) obtained through the integration of the pyroelectric current with no applied electric field in capacitive Si:HfO_2_ (9 nm) thin film structure in two different temperature ranges i.e. (**a**) 173–273 K and (**b**) 273–333 K in two steps, where Si doping is 5.6 mol% (reproduced with permission) [88].

In another report from Hoffmann et al., a promising enhancement in the PyEH in HfO_2_-based thin films was obtained [88]. Here, the temperature- and electric field-induced phase transitions were tuned by varying the Si doping (i.e., 3.8, 4.3, and 5.6 mol%) into an HfO_2_ thin film. A remarkable harvestable energy density of 20270 kJ∙m^−3^∙cycle^−1^ was obtained, as shown in Figure 6c, over a wide range of temperatures (while keeping the *T_L_* below room temperature, Δ*T* = 110–430 K and Δ*E* = 0–3330 kV∙cm^−1^) in 5.6 mol% Si:HfO_2_ thin films [88]. The Si:HfO_2_ thin films exhibited the phase transition from a pure ferroelectric to field-induced ferroelectric as the temperature increases, which can be exploited for the PyEH and ECE as well. The 5.6 mol% composition exhibited the strongest constriction in *P*–*E* loop at room temperature and highest temperature as well. A very high pyroelectric coefficient in Si:HfO_2_ thin films leads to the excellent *k*^2^ values as compared to other materials. Figure 7 shows the pyroelectric current for two different temperature ranges. The authors pointed out that the Hf_0.2_Zr_0.8_O_2_ thin films exhibited negligible pyroelectricity when no electric field is applied, hindering its application as sensors [87,88]. Meanwhile, in the case of the Si:HfO_2_ thin films, pyroelectricity is exhibited, even at zero applied electric field. Along with *k*^2^, the Si:HfO_2_ thin films possess *F_V_* values comparable to those of previously reported lead-based ceramics, such as Pb(Zr_x_Ti_1-x_)O_3_ (PZT) [112] and single-crystals of 0.75Pb(Mg_1/3_Nb_2/3_)O–0.25PbTiO_3_ (PMN-0.25PT) [113], while the (*F_i_*) values were slightly lower.

#### 3.2.2. Lead-Based Pyroelectric Materials

As we have discussed in the previous section, although some of the lead-free materials have exhibited superior thermal energy harvesting performance to that of the lead-based pyroelectric materials, the lead-based materials show superior FoMs for applications such as infrared sensors, which make them an excellent choice for commercial sensor application. In the quest for excellent materials for PyEH application, a plethora of studies have been reported on PZT- and PMN-PT-based ceramics and their thin films [64,85,108,114,115,116,117,118,119,120,121]. The phase transformations of the ceramics play a huge role in the PyEH energy density and efficiency. Various groups have studied temperature- or electric field-dependent phase transitions to enhance the PyEH ability of the ceramics. Jo et al. reported their study of the temperature-induced phase transformation of the (Pb_0.97_La_0.02_)(Zr_0.55_Sn_0.32_Ti_0.13_)O_3_ ceramics to enhance the PyEH ability. The authors obtained a maximum energy density of 270 kJ∙m^−3^∙cycle^−1^ from the ferroelectric material using the Olsen cycle with parameters Δ*T* = 298–453 K and Δ*E* = 10–80 kV∙cm^−1^ [92]. Similarly, the field-induced phase transitions of the (Pb,La)(Zr,Sn,Ti)O_3_ single-crystals fabricated with a composition close to the morphotropic phase boundary (MPB) were studied by Zhuo et al. [93]. Both aforementioned reports demonstrate the significant enhancement in the energy density of the PLZT-based bulk materials.

The enhanced pyroelectric properties with high FoMs were reported by Mangalam et al. in PZT [122]. In addition, La-doped PZT relaxor ferroelectrics have been explored previously for PyEH applications, in which doping yielded higher resistivity and pyroelectric coupling coefficient [108,123,124]. As we have discussed earlier, as compared to the bulk ceramics, thin films exhibited the more efficient PyEH, owing to its higher dielectric breakdown strength. Vats et al. reported the comparative study of the giant energy harvesting in 0.68PMN-0.32PT-based thin films with Pb(Zr_0.3_Ti_0.7_)O_3_/PbO_x_ buffer layer and <001>-oriented 0.67PMN-0.33PT thin films. In the calculation of the energy harvesting, the *T_L_* and *E_L_* are kept constant at 303 K and 0 kV∙cm^−1^. The obtained maximum energy densities for PMN-PT buffer-layered thin films and PMN-PT thin films were nearly 8000 kJ∙m^−3^∙cycle^−1^ (Δ*T* = 393 K and Δ*E* = 600 kV∙cm^−1^) and 6500 kJ∙m^−3^∙cycle^−1^ (Δ*T* = 393 K and Δ*E* = 600 kV∙cm^−1^), respectively. The notable point in this study was that there was a sharp jump in the energy density observed when the temperature range changes from 303 to 393 K to 303 to 423 K, which indicates that a further enhancement in the energy density can be achieved near its *T_c_* and MPB. The enhancement in the energy density (larger for PMN-PT buffered layer than the PMN-PT thin films) is attributed to the small difference in remnant polarization and low *T_c_* of the PMN-PT buffer layered thin films [94].

Although PLZT ceramics exhibit a lower pyroelectric coefficient than PMN-PT, the composition alterations of the antiferroelectric (AFE) PLZT ceramics exhibit promising harvestable energy density [108,113]. The AFE ceramics were reported with a high pyroelectric effect due to its field-induced phase transitions, which makes them potential candidate for the PyEH and refrigeration as well [108,125]. Hao et al. reported the maximum energy density of 7800 kJ∙m^−3^∙cycle^−1^ (Δ*T* = 298–558 K and Δ*E* = 300–900 kV∙cm^−1^) in the 2-µm-thick Pb_0.97_La_0.02_(Zr_0.75_Sn_0.18_Ti_0.07_)O_3_ AFE, which was the largest reported value up to that date [89]. Here, the energy density of the films was also calculated using the Olsen cycle. In addition, the calculated efficiency of the thermal energy conversion was reported ~0.53%, which is one order higher than that of general thermoelectrics. The energy density of the films was obtained at a 1-kHz frequency of the applied electric field. With the increase in the frequency of the applied electric field for the *P-E* hysteresis loop, the energy density value decreased. The authors obtained the maximum energy density of 8400 kJ∙m^−3^∙cycle^−1^ at 100 Hz. Therefore, it can be inferred that the thermal-electrical energy harvesting ability of the films can be optimized by optimizing associated parameters such as the operating temperature, applied electric field, and its frequency. In another report from the very same group, the ECE and PyEH characteristics were reported, as the authors thoroughly investigated the effects of the phase structure on the ECE and PyEH capacities [67]. Sn-doping into the 1.5-µm-thick (Pb_0.97_La_0.02_)(Zr_0.95−x_Sn_x_Ti_0.05_)O_3_ AFE films was varied with x = 0.08 (orthorhombic), 0.20 (tetragonal), and 0.38 (MPB) to control the phase structure, and maximum energy densities of 3800, 6800, and 4000 kJ∙m^−3^∙cycle^−1^ were achieved with x = 0.08, 0.20, and 0.38, respectively. The inference from the above two studies can be drawn that the PLZT-based AFE films yields a maximum energy density in the tetragonal phase.

**Figure 8:**
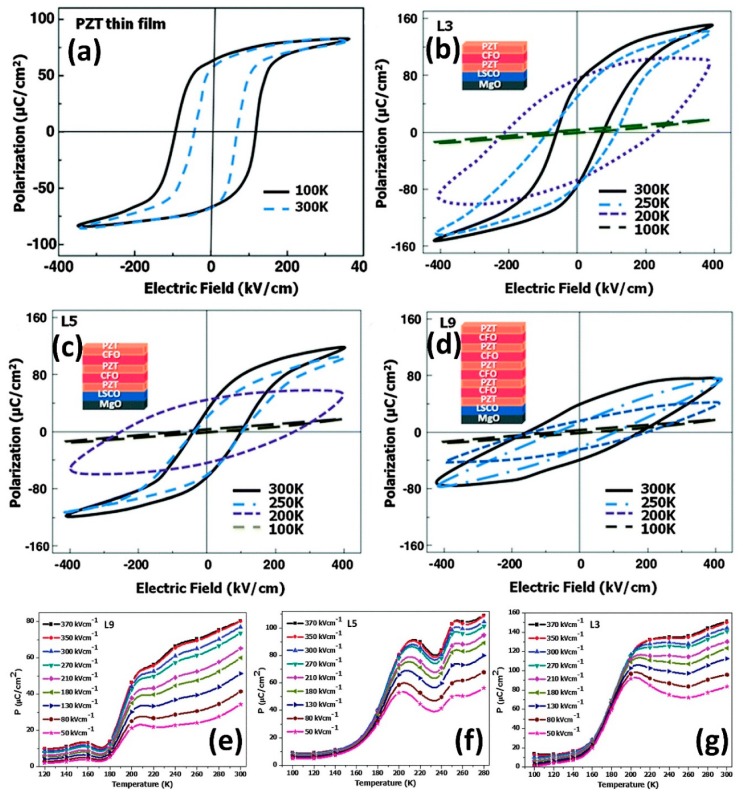
Temperature-dependent *P*–*E* hysteresis loops of PZT-CFO multilayer nanostructures, (**a**–**d**) for pure PZT thin films, L3, L5, and L9, respectively. The inset image shows the schematic of the multilayered nanostructured specimen. (**e**–**g**) show the temperature vs polarization plots for L3, L5, and L9, respectively, with different applied electric field. It can noted here that the polarization with regard to temperature (reproduced with permission) [82,126,127].

Apart from the thin and thick film structures, multilayer structures have been also explored. Vats et al. reported a remarkably giant PyEH per cycle of 47372 kJ∙m^−3^∙cycle^−1^ in PbZr_0.53_Ti_0.47_O_3_/CoFe_2_O_4_ (PZT/CFO) multilayered nanostructures (MLN) using the Olsen cycle, which was nearly four times higher than the highest reported PyEH up to the date of publication [82], as shown in Figure 8. The significant enhancement in the PyEH properties was attributed to the cumulative effects of the multilayers, which induce an enhancement in the polarization of the capacitive structure (~1.5 times ofPZT). As shown in Figure 8e–g the MLN structures exhibit a sharp increase in the polarization at approximately 200 K. The authors explained the enhancement in the polarization of the MLN structures is due to the dynamic magneto-electric coupling (MEC) [126,127]. The MEC effect is stronger in the low-temperature range and becomes weaker in the higher temperature range. In addition, it leads to an abrupt change in the polarization as the temperature fluctuates, i.e., dPs/dT is larger. In the study, the authors deposited a three-specimen, consisting of three (L3), five (L5), and nine (L9) alternating layers of PZT and CFO thin films on LSCO (100) coated MgO substrate using the pulsed laser deposition technique. Figure 8e–g shows the energy density plots for all the three configurations. The obtained maximum energy densities for these configurations were 23011 for (L9), 35278 for (L5), and 47372 (L3) kJ∙m^−3^∙cycle^−1^ (ΔT = 100–300 K and ΔE = 0–400 kV∙cm^−1^), which are higher than any reported value, as shown in Table 1. In the measurement of the energy density, the TL and EL are kept constant at 100 K and 0 kV∙cm^−1^, respectively.

## 4. Application of Pyroelectricity for Flexible Devices

Pyroelectric materials in bulk and thin-film forms have been discussed above for PyEH applications, such as heat sensors, thermal imaging or infrared sensors, fire alarms, and gas sensors. The selection of the materials is conducted based on the FoMs for these applications, i.e., *F_v_*, *F_i_*, *F_E_*, and *k*^2^, as these have already been discussed earlier in the above sections. This section will primarily focus on the applications of pyroelectric materials as energy harvesters and sensors.

### 4.1. Flexible Energy Harvesters

**Figure 9:**
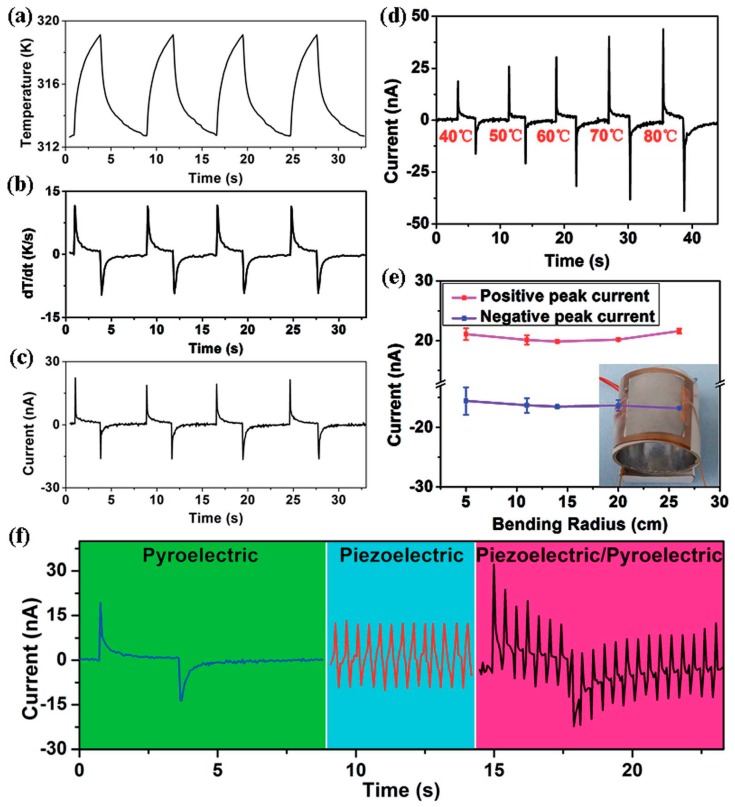
(**a**) Temperature change vs. time plot for the nanofiber membrane (NFM)-based nanogenerator, (**b**) the corresponding dT/dt plot, (**c**) the corresponding output current vs. time plot from the device, (**d**) measured output current of the nanogenerator when heated from different initial temperatures, (**e**) the output current from the nanogenerator when subjected to different mechanical stresses (varying the bending), and (**f**) the output current from the pyroelectric, piezoelectric, and simultaneous effects (hybrid) when subjected to temperature changes (heating and cooling for 3 s each) and mechanical stresses (f = 2.5 Hz), respectively (reproduced with permission) [37].

Among the reported PyEH devices, the flexible energy harvesting devices have generated much interest owing to its unlimited potential for applications such as wearable and plantable devices on human body [36,37,40,128,129]. Various ferroelectric materials such as PMN-PT, PLZT, and polymers (e.g., PVDF-TrFE) have been investigated and successfully demonstrated for such applications. Among these functional materials, polymers with their low cost and lightweight flexible and biocompatible properties are highly desired. For example, PVDF possesses both pyroelectric and piezoelectric properties, which make it an excellent choice for the fabrication of flexible hybrid energy cell. In addition, PVDF has attracted much interest in the scientific community for these applications because of its improved mechanical properties, geometrical effect, and high sensitivity for small mechanical stress or deformation. You et al. [37] demonstrated a nonwoven nanofiber membrane-based (comprising PVDF polymer) self-powered flexible hybrid (piezoelectric and pyroelectric) nanogenerator (Figure 9). The flexible hybrid nanogenerator structure comprises electrospun PVDF nanofiber membranes (NFMs), a thermoplastic polyurethane (TPU) NFM-carbon nanotube (CNT) composite and an electrospun poly(3,4-ethylenedioxythiophene):poly(styrene sulfonate)-polyvinyl pyrrolidone (PEDOT:PSS-PVP)-conductive NFM. The flexible TPU NFM-CNT was used as the substrate and the electrode as well to improve the flexibility of the device. For the flexible top electrode, PEDOT:PSS-PVP-conductive NFM was employed. The author examined the output voltage of the devices when they are subjected to mechanical stresses (compression and bending operations) and the temperature changes (cold and hot air flows) individually and simultaneously as well.

Zhang et al. [39] demonstrated a flexible PyNG based on a thin PVDF film. The measured output open circuit voltage and short circuit current were 8.2 V and 0.8 µA, respectively. The maximum output power was measured to be 2.2 µW with a load of 0.1 MΩ. The generated power can drive an LCD or LED or be stored in a capacitor for subsequent applications. Similarly, Yang et al. [41] also demonstrated a flexible hybrid energy harvesting cell consisting of a pyroelectric NG, piezoelectric NG, and solar cell, which can be employed to individually or simultaneously harvest thermal, mechanical, and solar energies. The presented cell was fabricated using PVDF film and flexible ZnO nanowire array- poly(3-hexylthiophene) P3HT film heterojunctions (for scavenging the solar energy). The output energy from the energy harvester was stored in an Li-ion battery, which can drive four LEDs in parallel connection. Thus, the proposed cells have great potential in various applications such as wireless sensor systems, environmental surveillance, medical diagnostics, and defense technologies. Chen et al. [45] reported flexible hybrid piezoelectric–pyroelectric NG based on PVDF-TrFE with improved output voltage/current (4.0 V/65 nA when subjected to mechanical bending and 3.2 V/52 nA when subjected to temperature change by heat–cool operations with a temperature range of 8 K near room temperature). Recently, Zhao et al. [130] enhanced the output voltage and current of the PVDF-based flexible PyEH for the self-powered temperature monitor of chemical exothermic processes applications. The obtained output voltage and current (with a load of 100 MΩ) were 9.1 V and 95 nA, respectively.

**Figure 10:**
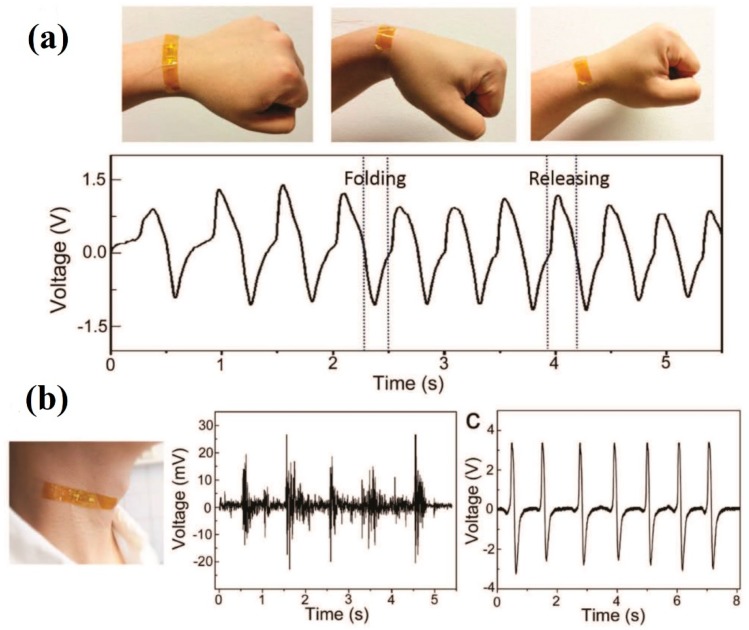
(**a**) Image of the PMN-PT ribbon-based device attached on the wrist skin. The plots below the image show the output voltage obtained while folding and resealing actions of the wrist. (**b**) Image of the attached device on the neck and subsequent plot show the output voltage waveforms of the device generated from coughing, and (**c**) shows the output voltage generated from small stick knocking (reproduced with permission) [36].

**Figure 11:**
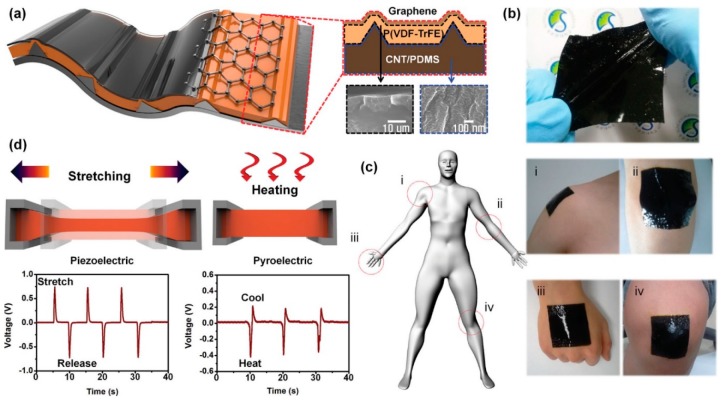
(**a**) Schematic representation of the flexible hybrid energy harvester, (**b**,**c**) images of the hybrid stretchable nanogenerator (HSNG) employed at various places on human body, and (**d**) piezoelectric and pyroelectric output voltages from the HSNG when subjected under stretch–release and cool–heat (thermal gradient) conditions (reproduced with permission) [43].

Apart from the polymer-based energy harvesters, lead-based and lead-free ceramic thin films have also been explored owing to their excellent pyroelectric properties [36,40,42,44]. Chen et al. demonstrated a flexible PMN-PT ribbon-based piezoelectric and pyroelectric hybrid generator to scavenge the mechanical movement of human body parts and temperature monitoring, as shown in Figure 10. It consists of micropatterned single-crystal PMN-PT ribbons. The flexible PMN-PT ribbon-based sensor was conformally attached onto the surface of the human skin, which enables high sensitivity to human body motion. It also can be used to precisely detect acoustic sounds. The implanted sensor can be used to monitor temperature-related activity. The generated output voltage waveforms of the device from the mechanical movements of human body have been shown in Figure 11. Although the perovskites have exhibited excellent piezoelectric and pyroelectric coefficients, the energy harvesters based on these materials have not been significantly explored. A flexible and hybrid pyroelectric–piezoelectric NG based on Pb(Zr_0.52_Ti_0.48_)O_3_ films was examined by Ko et al. [44]. To fabricate the flexible device, The PZT thin film was deposited on highly flexible Ni–Cr metal foil substrate, with LaNiO_3_ as the bottom electrode, which enables the high temperature growth of the film. The flexible PZT film possesses high piezoelectric (140 pC/N) and pyroelectric coefficients (50 nC/cm^2^∙K) at room temperature. Among the aforementioned polymers and lead-based PyEHs, the lead-free material is also highly desired for flexible energy harvesting applications owing to its high-temperature stability and environmental compatibility. Yang et al. [42], demonstrated the flexible pyroelectric NGs consisting of a composite structure of lead-free KNbO_3_ nanowires-PDMS.

### 4.2. Hybrid Harvester with Pyroelectric Materials (Nano-Generators)

PyEH efficiency can be enhanced by employing various energy sources. Basically, the hybrid energy harvesters are designed by employing two or more energy conversion mechanisms, such as pyroelectric, piezoelectric, photothermal, and thermoelectric processes [131], while ensuring that each coupled harvesting method does not restrain the others. In this section, we will be focusing on the hybrid energy harvesters consisting of pyroelectric and piezoelectric systems.

Because all the pyroelectric materials are also piezoelectric materials, there was great interest in the scientific community to attempt to combine both energy harvesting methods. In addition, there is a close similarity between the relevant equations of the pyroelectricity and piezoelectricity, as the temperature change Δ*T* is analogous to the stress Δ*σ* for the piezoelectricity. Owing to the analogous symmetry of these two energy harvesting methods, much effort has been put into designing hybrid pyroelectric–piezoelectric energy harvesting systems [36,37,41,43,44,45,132]. In such hybrid systems, the changes in the polarization should be constructive and further enhance the power generation of the energy harvester.

Lee et al. [43] reported a highly stretchable pyroelectric–piezoelectric hybrid nanogenerator consisting of micropatterned PDMS–carbon nanotube (CNT) composites, micropatterned piezoelectric PVDF-polytrifluoroethylene (PTrFE) polymer, and graphene nanosheets, as shown in Figure 11. At the base of the device, to make it flexible, PDMS-CNTs were employed. For the top flexible electrode, graphene was employed, which has high thermal conductivity, thus allowing for fast temperature sensing on the device. The authors demonstrated the potential of the material for harvesting temperature difference (Δ*T*) and mechanical stress (*σ*). Here, the cumulative change in the polarization can be expressed as

(12) ΔP=d·σ+λ·ΔT

where *d* is the piezoelectric coefficient.

Wang et al. [133] demonstrated the hybrid nanogenerator, consisting of a PVDF nanowire-PDMS composite/indium tin oxide (ITO)/PVDF/ITO structure. The presented harvesting device is demonstrated to be able to scavenge thermal and mechanical energies individually or simultaneously using pyroelectric, piezoelectric, and triboelectric effects. Erturun et al. [119] investigated the hybrid energy harvester employing a heating lamp directed at a vibrating beam. In the study, both effects (i.e., mechanical and thermal energy harvesting) were examined individually and combined later. The authors observed that in some cases, the beam vibration with thermal cycling inhibited the energy conversion performance of the device and placed a negative effect resulting from the difference in the frequencies of the temperature cycles and mechanical vibrations. Although there are certainly some factors which limiting the cumulative performance of the hybrid energy harvesters, the use of pyroelectric–piezoelectric hybrid energy harvesters has great potential to enhance the output power.

### 4.3. Flexible Sensors

Since the last few decades, small and wearable electronic devices have received enormous attention. Correspondingly, several pyroelectric materials have been studied and employed commercially for the sensor applications. A human body maintains its certain temperature and continuously emits infrared rays. Therefore, especially in the winter, there is a temperature gradient between human body and surrounding environment which can be further exploited to generate the electric potential. So, if PyNGs are placed in the human body in form of wearable devices, the temperature fluctuations can be converted into the electric potential between electrodes due to pyroelectric effect. Especially in winter, due to cold weather or air pollution, people prefer to wear a respirator; the human body temperature is ~37 °C, while for some regions with high altitude, the outdoor temperature in winter is as low as −20 to −30 °C. Xue et al. demonstrated the self-powered respiratory system installed with the proposed PyNG [46]. Similarly, PVDF was also demonstrated as temperature sensors by Pullano et al. [134,135].

High FoMs, such as *F_v_* and *F_i_*, are of importance while choosing the materials for sensors applications. The studies have suggested that triglycine sulfide (TGS) has great potential for pyroelectric energy conversion applications. Although the TGS has exhibited excellent FoMs, its application arena is limited because of its low Curie temperature (T_c_ = 49 °C) [76]. A few lead-based ceramics such as PMN-PT and PZT have been demonstrated with excellent FoMs for pyroelectric energy conversion applications [34,53,59,115,117,120,121,136,137]. Similarly, several lead-free ceramic pyroelectric materials such as BNT-BT [138], barium strontium titanate (BST) [139], potassium sodium niobite (KNN) [140,141] have also been reported with excellent FoMs.

To obtain the best performance from the pyroelectric material, there is a tradeoff between the pyroelectric coefficient, dielectric loss, dielectric constant, and material specific heat, which further limits the enhancement of the FoMs. Researchers have explored the alteration in the chemical and physical composition of ceramics, such as doping or introduction of pores into the ceramics [142,143,144]. For such purposes, methods such as partial sintering and adding pore former agents have been explored. Several pore formers, e.g., poly(methyl methacrylate) (PMMA), carbon nanotubes (CNT), and ethyl cellulose, have been employed into ceramics, such as Pb(Zr_0.965_Ti_0.035_)O_3_ + 1 wt.% Nb_2_O_5_, BaSn_0.05_Ti_0.95_O_3_, Ba_0.67_Sr_0.33_TiO_3_, and PbZr_0.45_Ti_0.55_O_3_ [144,145,146,147,148]. These studies demonstrated that the enhancement in the porosity of the ceramics resulted in the improvement in the FoMs [148,149].

## 5. Conclusions and Future Outlooks

In this review, the recent progress in pyroelectric ceramics/polymers, its thick and thin films for flexible energy harvesting, sensors, and as hybrid piezoelectric–pyroelectric applications in addition to the fundamental theories on pyroelectricity is systematically discussed. The discussion primarily was focused on the PyEHs where the measurement and calculations of the maximum harness-able energy density was done using Olsen cycle. The thermal energy harvesting performance of various lead-based, lead-free polymers and their prospects has been highlighted. The discussed studies in this article provide broad applications of the pyroelectric materials as PyEH and heat sensing devices. As the hybrid energy harvesters have a great potential as flexible and wearable energy harvesting devices (as shown in Figure 9, Figure 10 and Figure 11). Furthermore, it concluded that the maximum harness-able energy density, output voltage and current and efficiency are highly dependent on material selection and structures. As for the fabrication and application point of view, the PyEH devices are easier in structure so easy to fabricate. Regarding the potential pyroelectric harvesting cycles, the implementation of the pyroelectric element is easy and can operate in broad range of temperature (until and unless the material loses its polar nature). The more significant efforts towards the designing of PyEHs at the nanoscale would be great as it may bring opportunities for high frequency applications. In order to obtain the high output power, the pyroelectric materials need high pyroelectric coefficient in operating temperature range. Moreover, in order to obtain huge energy conversion using Olsen cycle, the pyroelectric materials should possess a huge difference in *P_s_* vs. temperature change (*dP_s_/dT*), a low piezoelectric coefficient (since higher coefficient causes piezoelectric noise in the device), the *T_c_* should be near to operating temperature, and the material should have low dielectric loss and a high dielectric constant. Also, the pyroelectric material must possess the high resistivity and low coercive field. As the pyroelectric materials have shown higher performance near the MPB, there have been several efforts to engineer the materials (altering the compositions or forming the composites) to obtain the desired phase structure. Apart from these, the introduction of lower permittivity material into the pores of the pyroelectric material ceramic has helped significantly in enhancing its pyroelectric energy harvesting performance.

The PyEH devices are currently limited by the inability to induce high frequency temperature fluctuations which further limits the harvestable amount of power. Similarly, in case of the hybrid pyroelectric–piezoelectric energy harvesters, the output power is largely restrained by the difference in the frequency of the mechanical stresses (bending or vibrations) and temperature changes (i.e., *dT/dt*). However, the more optimized paradigms of the hybrid PyEH might be quite helpful in enhancing the output power.

## Figures and Tables

**Table 1:** Recently reported materials for the pyroelectric thermal energy harvesting using the Olsen cycle (TF = Thin film; BC = Bulk ceramic; TF’ = Thick film; SC = Single-crystal; a = the shown values are estimated from reported data).

Materials	Form	*T_low_* (K)	*T_high_* (K)	*E_low_* (kVcm^−1^)	*E_high_* (kVcm^−1^)	Maximum Harvestable Energy Density (kJm^−3^ cycle^−1^)	Ref.
PbZr_0.53_Ti_0.47_O_3_/CoFe_2_O_4_	TF	100	300	0	400	47,372	[82]
K[(Nb_0.90_Ta_0.10_)_0.99_Mn_0.01_]O_3_	BC	413	433	1	50	629	[83]
0.74Na_0.5_Bi_0.5_TiO_3_-0.06BaTiO_3_-0.20SrTiO_3_	BC	303	423	0	40	425	[68]
(Bi_0.5_Na_0.5_)_0.915_(Bi_0.5_K_0.5_)_0.05_ Ba_0.02_Sr_0.015_TiO_3_	BC	293	433	1	40	1523	[84]
Pb(Zr_x_Ti_1-x_)O_3_	BC	-	-	1	25	348	[85]
Bi_0.5_Na_0.44_K_0.06_TiO_3_	BC	298	393	1	52	1986	[86]
Hf_0.2_Zr_0.8_O_2_	TF	298	423	0	3260	11,500	[87]
5.6 mol% Si:HfO_2_	TF	110 ^a^	430 ^a^	0 ^a^	3330 ^a^	20,270	[88]
Pb_0.97_La_0.02_(Zr_0.75_Sn_0.18_ Ti_0.07_)O_3_	TF’	298	558	300	900	7800	[89]
(Pb_0.97_La_0.02_)(Zr_0.75_Sn_0.20_ Ti_0.05_)O_3_	TF’	293	473	300	900	6800	[67]
Pb_0.99_Nb_0.02_(Zr_0.55_Sn_0.40_ Ti_0.05_)_0.98_O_3_	TF	298	498	218	1091	7350	[90]
[111]-Oriented (Pb_0.967_La_0.022_)(Zr_0.64_Sn_0.23_Ti_0.13_)O_3_	SC	298	453	5	30	620	[91]
(Pb_0.97_La_0.02_)(Zr_0.55_Sn_0.32_ Ti_0.13_)O_3_	BC	298	453	10	80	270	[92]
Pb_0.97_La_0.02_Zr_0.57_Sn_0.24_Ti_0.19_O_3_	SC	298	448	0	30	400	[93]
0.68PbMg_1/3_N_2/3_O_3_–0.32PbTiO_3_	TF	303	323	0	600	8000	[94]
